# Histological evaluation of palatal donor site healing with leukocyte–platelet–rich fibrin versus collagen sponge

**DOI:** 10.4317/jced.63488

**Published:** 2025-12-30

**Authors:** José C. Rosas-Díaz, María E. Guerrero, Nancy E. Córdova-Limaylla, César-Augusto Padilla-Avalos, Jerson J. Palomino-Zorrilla, Rocio del Carmen Alvarez-Medina

**Affiliations:** 1 School of Stomatology, Universidad Privada San Juan Bautista, Lima, Perú; 2 Department of Medico Surgical Stomatology, Faculty of Dentistry, Universidad Nacional Mayor de San Marcos, Lima, Perú; 3 Research Professor, Research and Innovation Laboratory in Digital Dentistry, School of Dentistry, Universidad Científica del Sur, Lima, Perú; 4 School of Dentistry, Universidad Científica del Sur. Lima, Perú

## Abstract

**Background:**

The palate is the primary donor site for autogenous connective tissue grafts in periodontal and peri-implant plastic surgery, yet healing by secondary intention often results in morbidity. Collagen sponge (CS) and leukocyte-platelet-rich fibrin (L-PRF) have been proposed to enhance donor site repair, but comparative histological evidence in humans remains scarce. Objective: To compare the histological characteristics of palatal donor site healing following coverage with CS or L-PRF.

**Material and Methods:**

A retrospective cross-sectional histological study was performed on palatal biopsies collected four months after connective tissue graft harvesting covered with CS (n = 9) or L-PRF (n = 9). Epithelial type and thickness, lamina propria thickness, submucosal composition, inflammatory infiltrate, vascular congestion, and edema were evaluated using hematoxylin-eosin staining.

**Results:**

Both biomaterials supported uneventful healing without necrosis or severe inflammation. Compared with CS, L-PRF was associated with thicker epithelium, a higher frequency of hyperparakeratinization, and the presence of orthokeratinization. Lamina propria thickness was slightly greater in L-PRF, while fibrous submucosa predominated in both groups. Mild leukocyte infiltration and transient edema were more common with L-PRF, suggesting a more active regenerative response.

**Conclusions:**

CS and L-PRF both promoted favorable palatal donor site healing. L-PRF demonstrated histological features consistent with enhanced tissue regeneration, likely due to its growth factor content. These preliminary findings warrant validation in randomized controlled trials with larger sample sizes.

## Introduction

Obtaining autologous soft tissue grafts, either as free gingival grafts or subepithelial connective tissue grafts, remains the gold standard in periodontal and peri-implant plastic surgery due to its predictable effectiveness in augmenting keratinized tissue thickness and improving gingival esthetics around both teeth and implants. The palate remains the preferred donor site for these procedures (1 - 4). However, harvesting free gingival grafts creates a secondary intraoral wound that heals by secondary intention, a process frequently associated with morbidity (5). Palatal wound healing progresses through sequential phases of hemostasis, inflammation, proliferation, and remodeling. The initial inflammatory phase involves coagulation and leukocyte infiltration, followed by angiogenesis, fibroblast migration, and reepithelialization during proliferation. Remodeling reorganizes the extracellular matrix and restores functional integrity (3). Despite this orchestrated sequence, postoperative morbidity-characterized by pain and a healing process that can extend up to 12 weeks-may be exacerbated by microbial load and persistent inflammatory mediators, compromising epithelial and connective tissue regeneration (6 , 7). To mitigate these drawbacks, biomaterials such as collagen sponges (CS) and autologous platelet concentrates, particularly leukocyte-platelet-rich fibrin (L-PRF), have been proposed as adjuncts to accelerate healing and modulate the tissue environment (4). CS acts as a resorbable scaffold that stabilizes the clot, facilitates cellular migration, and promotes type I collagen synthesis (8 , 9). In contrast, L-PRF is a second-generation autologous platelet concentrate that combines platelets, leukocytes, and a dense fibrin network, enabling the sustained release of growth factors. This biomaterial has demonstrated the ability to stimulate angiogenesis, enhance cell proliferation, and promote tissue remodeling, while also reducing postoperative discomfort (5 , 10). Histomorphological evaluation of palatal mucosa provides valuable insights into the quality of regenerated tissue, including epithelial architecture, collagen fiber arrangement, vascularization, and residual inflammatory activity (11 , 12). Although several clinical studies and systematic reviews have investigated CS and L-PRF in palatal wound management (4 , 5 , 7 , 13), comparative histological evidence in humans remains scarce, particularly regarding medium-term healing of the palatal donor site. This knowledge gap is relevant because the histological quality of regenerated mucosa not only influences patient morbidity but also determines the availability of future donor tissue. Therefore, the present study aimed to compare, through histological analysis, the quality of palatal donor site healing covered with CS or L-PRF, focusing on parameters such as epithelial differentiation and thickness, lamina propria density, inflammatory infiltrate, vascular response, and edema.

## Material and Methods

**Figure 1:**
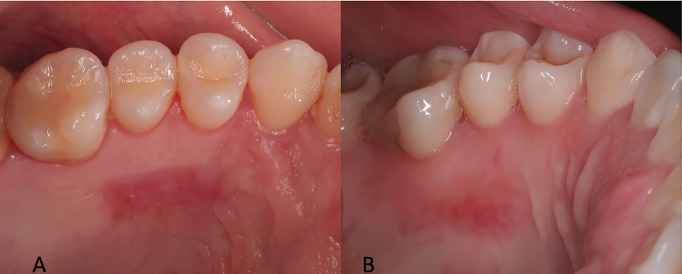
Representative clinical images of palatal donor site healing at day 28. (A)Site covered with a collagen sponge. (B)Site covered with leukocyte–platelet–rich fibrin (L-PRF). Complete epithelialization was observed in both cases without complications.

**Figure 2:**
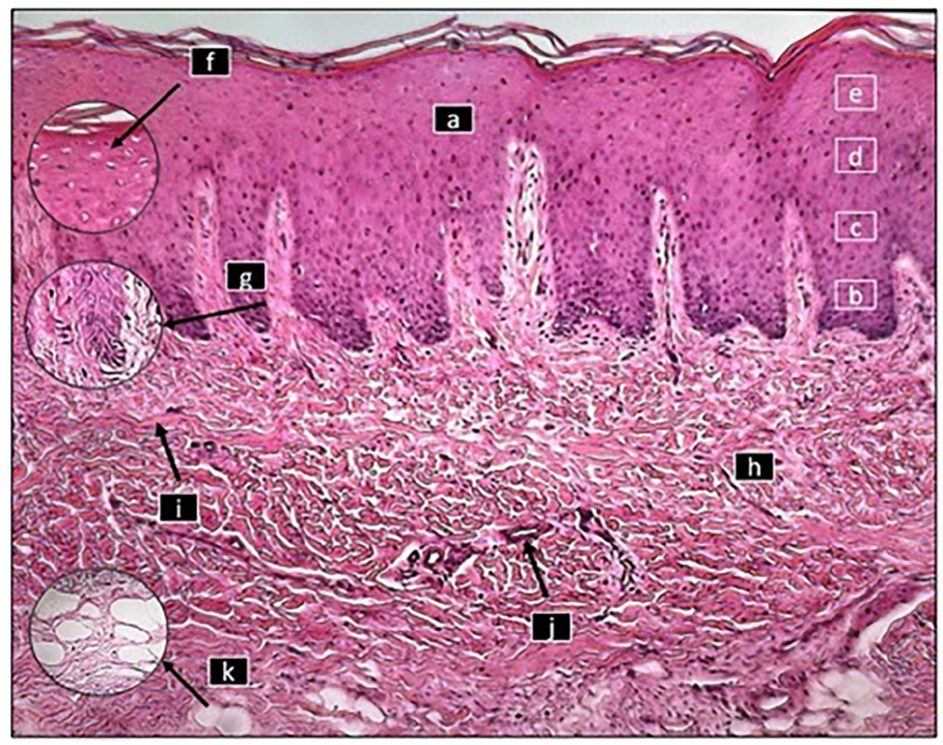
Histological section of the mucosa of the healed palate with PRF where the epithelium (a) with its basal (b), spinous (c), granular (d) and corneal (e) strata can be seen at 8X magnification, as well as some of the cells of the stratum corneum that have a nucleus (f) as well as cylindrical cells with normal characteristics in the basal stratum (g). In the connective tissue (h), the presence of collagen fibers (i), blood vessels (j), as well as adipose tissue with normal characteristics can be observed. (k)

- Ethical Approval and Informed Consent This study represents a secondary analysis derived from a previously published investigation approved by the Research Ethics Committee of Universidad Privada San Juan Bautista (Approval code: 891-2021-CIEI-UPSJB). Written informed consent was obtained from all participants, authorizing the use of their clinical data and biological samples for research purposes. In some patients requiring additional connective tissue grafting, small supplementary palatal samples were collected for histological evaluation, without adding risk beyond routine care. All procedures adhered to the principles of the Declaration of Helsinki and national data protection regulations. - Study Design A retrospective cross-sectional histological study was conducted based on palatal tissue samples obtained during connective tissue graft procedures for gingival defect reconstruction. The primary objective was to evaluate histological characteristics of palatal wound healing at four months post-harvest, according to the biomaterial used for donor site coverage (collagen sponge [CS] or leukocyte-platelet-rich fibrin [L-PRF]). - Population and Sample Patients were treated at Clínica Watanabe & Cadena (Lima, Peru) between September 2021 and March 2022. Inclusion criteria were: systemically healthy adults (American Society of Anesthesiologists [ASA I]), aged 18 years, with no active dental or periodontal pathology, oral hygiene index 20%, and requiring periodontal or peri-implant surgery with connective tissue grafting. Exclusion criteria included smoking, pregnancy, systemic disease, coagulation disorders, medications affecting wound healing, or hypersensitivity to the study materials. The final sample consisted of 18 patients (10 women and 8 men), equally divided into CS (n = 9) and L-PRF (n = 9) groups. Patient allocation was non-randomized, based on clinical availability during the study period. The sample size was determined by patient availability, given the exploratory nature of the analysis. - Surgical Procedures All surgical interventions were performed by a periodontics specialist. Following initial prophylaxis and plaque control, gingival health was confirmed. Extraoral antisepsis was carried out with povidone-iodine and intraoral antisepsis with 0.12% chlorhexidine. Connective tissue was harvested using a free palatal graft technique. Donor sites were then covered with either a collagen sponge (Hemotamp, Lima, Peru) or a leukocyte-platelet-rich fibrin (L-PRF) clot, prepared by centrifugation at 13,000 rpm for 8 minutes using a Choukroun A L-PRF 12 centrifuge (France), in accordance with the manufacturer's protocol. Fibrin clots were compressed into membranes using the L-PRF Box system prior to placement. Standard postoperative follow-ups were conducted at 24 hours and at 7, 14, 21, and 28 days. The clinical healing of palatal donor sites, including photographs and clinical outcomes (epithelialization time, postoperative discomfort, and complications), was previously reported in an earlier publication using the same patient cohort (4). In summary, all donor sites showed complete epithelialization by day 28 without complications (Figs. 1,2).

1,2

Therefore, the present study focuses exclusively on the histological evaluation of tissue regeneration and does not duplicate the previously published clinical findings. - Timing and Rationale for the Biopsy Some patients presented with multiple gingival recessions requiring staged surgical interventions. Four months after the initial connective tissue harvesting-when complete clinical healing of the palatal donor site had occurred-a second surgical procedure was planned to obtain an additional graft. This strategy allowed us to obtain biopsy samples without altering the therapeutic protocol, taking advantage of a clinically necessary second surgery in patients requiring multiple grafting procedures. During this second intervention, a 5 mm punch biopsy was performed on the previously treated palatal area, extending down to the periosteum. The purpose of this biopsy was to evaluate the histological characteristics of palatal wound healing, depending on the biomaterial (CS or L-PRF) used to cover the donor site during the first procedure. This evaluation allowed for direct comparison of tissue regeneration between the two materials in human oral mucosa under real clinical conditions. The biopsy was incorporated into the second graft-harvesting procedure and did not alter the treatment plan. - Histological Processing and Evaluation All tissue samples were fixed in 10% formalin, embedded in paraffin, sectioned, and stained with hematoxylin-eosin using standard protocols. One histological slide (two sections) per biopsy was analyzed by an experienced oral pathologist using a Leica® optical microscope at 10× and 40× magnification. The following parameters were assessed: epithelial thickness (from the corneal surface to the basement membrane), lamina propria thickness (from the basement membrane to the submucosa), epithelial type (parakeratinized, hyperparakeratinized, or orthokeratinized), submucosal composition (fibrous or adipose), inflammatory infiltrate (absent, minimal, mild), vascular congestion (absent, minimal, mild, moderate), and edema (absent, minimal, mild, moderate). - Statistical Analysis Data were analyzed using IBM SPSS Statistics (v27.0; IBM Corp., Armonk, NY, USA). Descriptive statistics were calculated for all variables. Continuous variables were expressed as mean, standard deviation, median, and range; categorical variables as frequencies and percentages. Comparisons between CS and L-PRF groups were exploratory, emphasizing clinically relevant patterns to support hypothesis generation for future studies with greater statistical power.

## Results

A total of 18 patients were included, equally distributed between the collagen sponge (CS, n = 9) and leukocyte-platelet-rich fibrin (L-PRF, n = 9) groups. The mean age was 45.3 ± 11.2 years (range, 28-61), and 55.6% of participants were female. Histologically, CS specimens predominantly exhibited parakeratinized epithelium (66.7%), whereas L-PRF specimens showed a higher frequency of hyperparakeratinized epithelium (66.7%), with orthokeratinization observed exclusively in the L-PRF group (11.1%) (Table 1).

Table 1

Fibrous submucosa was the most common connective tissue type in both groups, although adipose tissue was slightly more frequent in L-PRF (44.4% vs. 33.3%). Quantitative analysis revealed that epithelial thickness was greater in the L-PRF group (0.446 ± 0.152 mm) than in the CS group (0.348 ± 0.114 mm). Similarly, lamina propria thickness was slightly higher with L-PRF (3.138 ± 0.454 mm) than with CS (3.062 ± 0.377 mm) (Table 2).

Table 2

Regarding tissue integrity and inflammatory response, both groups showed predominantly normal epithelium, with focal erosion or mild cellular degeneration in a minority of specimens. Leukocyte infiltration was absent or minimal in most cases, although mild infiltration was observed only in L-PRF samples. Vascular congestion was generally moderate in both groups, while mild edema was more frequent in L-PRF (75%) compared with CS (50%) (Table 3).

Table 3

## Discussion

Autologous soft tissue grafting remains fundamental for periodontal and peri-implant reconstructive procedures, with free gingival and subepithelial connective tissue grafts demonstrating predictable outcomes in augmenting gingival thickness and width of keratinized tissue (1 , 2 , 14 , 15). Nevertheless, donor site morbidity, including pain, inflammation, and delayed healing, continues to motivate the exploration of biomaterials, such as collagen sponges (CS) and leukocyte-platelet-rich fibrin (L-PRF), as adjunctive strategies to enhance palatal repair (4 , 16).

In this study, both CS and L-PRF demonstrated favorable biocompatibility, with no evidence of necrosis or severe inflammation. However, distinct epithelial patterns were observed: CS was associated with a predominance of parakeratinized epithelium, while L-PRF favored hyperparakeratinization and uniquely induced orthokeratinization. These findings may be attributed to the release of bioactive molecules in L-PRF, including platelet-derived growth factor (PDGF) and transforming growth factor- (TGF-), which are known to stimulate epithelial proliferation and remodeling (5 , 10 , 17). Similar trends have been confirmed in recent randomized trials and umbrella reviews, which report accelerated epithelialization and improved patient-reported outcomes with L-PRF compared to conventional dressings (6 , 7 , 19).

The vascular profile also differed between groups. L-PRF specimens demonstrated a higher frequency of mild vascular congestion and edema, findings that may reflect early angiogenic stimulation mediated by vascular endothelial growth factor (VEGF) and nitric oxide pathways (18 , 20). Although these features could be interpreted as transient inflammatory changes, they are consistent with the physiological remodeling phase of wound healing. Recent clinical data suggest that this angiogenic activity contributes to faster mucosal healing and reduced donor-site morbidity (5 , 19).

By contrast, CS primarily provided a passive scaffold, supporting fibroblast migration and extracellular matrix deposition without actively modulating cellular differentiation or angiogenesis (8 , 9). This may explain the absence of orthokeratinization in the CS group. Nevertheless, CS remained effective as a biocompatible covering material, consistent with findings from other histological and clinical studies showing satisfactory, albeit slower, mucosal repair (11 , 12 , 21 , 22).

The submucosal findings in both groups, with a predominance of fibrous connective tissue and variable adipose content, mirror prior histological studies that describe high interindividual variability in palatal tissue composition (11 , 12 , 22). Importantly, this variability did not appear to compromise healing quality when adequate vascularization was preserved.

Taken together, the results indicate that both CS and L-PRF are safe and effective for palatal donor site coverage, but L-PRF may elicit a more active regenerative response, characterized by increased epithelial thickness, altered keratinization, and increased angiogenic activity. These histological features could translate into clinically meaningful benefits, particularly in cases where accelerated healing and improved donor tissue quality are desirable.

Limitations of this study include the small, non-randomized sample and the reliance on a single histological section per biopsy, which may limit representativeness. In addition, the exploratory design precludes definitive statistical inference. Future research should include multicenter randomized controlled trials with standardized protocols, larger sample sizes, and longitudinal follow-up to validate these preliminary findings.

Clinical significance: Within the limitations of this study, L-PRF appears to enhance palatal donor site healing at the histological level, potentially reducing morbidity and improving the quality of regenerated mucosa for future graft harvesting.

## Figures and Tables

**Table 1:** Distribution of epithelium and submucosa types according to biomaterial (CS vs L-PRF).

Epithelium	Hyperparakeratinized	3 (33.3)	6 (66.7)
	Orthokeratinized	–	1 (11.1)
	Parakeratinized	6 (66.7)	2 (22.2)
Submucosa	Adipose	3 (33.3)	4 (44.4)
	Fibrous	6 (66.7)	5 (55.6)

CS: collagen sponge; L-PRF: leukocyte–platelet rich fibrin.

**Table 2:** Epithelial and lamina propria thickness (mm) according to biomaterial type.

Epithelial thickness	CS	0.348 (0.114)	0.343	0.130	0.511
	L-PRF	0.446 (0.152)	0.490	0.183	0.642
Lamina propria thickness	CS	3.062 (0.377)	3.08	2.41	3.49
	L-PRF	3.138 (0.454)	3.23	2.52	3.76

CS: collagen sponge; L-PRF: leukocyte–platelet rich fibrin.

**Table 3:** Distribution of histological inflammatory characteristics according to biomaterial type (CS vs L-PRF).

Epithelium alteration	Normal, intact	5 (50)	4 (50)
	Focal erosion	4 (40)	3 (37.5)
	Cellular degeneration, tissue flattening	1 (10)	1 (12.5)
Leukocyte infiltration	Absent	7 (70)	5 (62.5)
	Minimal	3 (30)	2 (25)
	Mild	-	1 (12)
Vascular congestion	Minimal	2 (20)	1 (12)
	Mild	2 (20)	3 (37.5)
	Moderate	6 (60)	4 (50)
Edema	Absent	1 (10)	1 (12.5)
	Minimal	4 (40)	1 (12.5)
	Mild	5 (50)	6 (75)

CS: collagen sponge; L-PRF: leukocyte–platelet rich fibrin.

## Data Availability

The datasets used and/or analyzed during the current study are available from the corresponding author.
